# Telomeres in Plants and Humans: Not So Different, Not So Similar

**DOI:** 10.3390/cells8010058

**Published:** 2019-01-16

**Authors:** Petra Procházková Schrumpfová, Miloslava Fojtová, Jiří Fajkus

**Affiliations:** 1 Laboratory of Functional Genomics and Proteomics, National Centre for Biomolecular Research, Faculty of Science, Masaryk University, CZ-61137 Brno, Czech Republic; petra.proch.schrumpfova@gmail.com (P.P.S.); miloslava.fojtova@gmail.com (M.F.); 2 Mendel Centre for Plant Genomics and Proteomics, CEITEC, Masaryk University, CZ-62500 Brno, Czech Republic; 3 Institute of Biophysics of the Czech Academy of Sciences, CZ-61265 Brno, Czech Republic

**Keywords:** telomere, telomerase, human, *Arabidopsis*, aging, chromatin, epigenetics, review

## Abstract

Parallel research on multiple model organisms shows that while some principles of telomere biology are conserved among all eukaryotic kingdoms, we also find some deviations that reflect different evolutionary paths and life strategies, which may have diversified after the establishment of telomerase as a primary mechanism for telomere maintenance. Much more than animals, plants have to cope with environmental stressors, including genotoxic factors, due to their sessile lifestyle. This is, in principle, made possible by an increased capacity and efficiency of the molecular systems ensuring maintenance of genome stability, as well as a higher tolerance to genome instability. Furthermore, plant ontogenesis differs from that of animals in which tissue differentiation and telomerase silencing occur during early embryonic development, and the “telomere clock” in somatic cells may act as a preventive measure against carcinogenesis. This does not happen in plants, where growth and ontogenesis occur through the serial division of apical meristems consisting of a small group of stem cells that generate a linear series of cells, which differentiate into an array of cell types that make a shoot and root. Flowers, as generative plant organs, initiate from the shoot apical meristem in mature plants which is incompatible with the human-like developmental telomere shortening. In this review, we discuss differences between human and plant telomere biology and the implications for aging, genome stability, and cell and organism survival. In particular, we provide a comprehensive comparative overview of telomere proteins acting in humans and in *Arabidopsis thaliana* model plant, and discuss distinct epigenetic features of telomeric chromatin in these species.

## 1. Introduction

Telomere biology, whose foundations were laid out in maize and *Drosophila* at the end of the 1930s and which developed at the molecular level in the 1980s, has flourished enourmously in the last 30 years. This interest in telomere biology follows from the generally attractive links between telomere functions, cell aging mechanisms, and the genesis of severe diseases in humans. Research in recent decades has elucidated the principles of protection of the ends of linear eukaryotic chromosomes from progressive shortening due to the incomplete replication (end-replication problem) [1] and from their erroneous recognition as unrepaired chromosome breaks (end-protection problem) [2,3,4]. In addition to these basic functions, other potential roles of telomeres have been suggested, such as a trap for reactive oxygen species [5,6]. Telomeres are composed of non-coding repetitive tandem repeats of (TTAGGG)_n_ in humans and the other vertebrates, and (TTTAGGG)_n_ in most plants. During human aging, telomeres in most somatic cells are shortened at each cell division and it is generally assumed that when telomeres reach a critical length, cells enter a senescent state and cell division ceases [7,8]. However, most human individuals do not reach this critical telomere length brink during their life course [8,9], e.g., the mean leukocyte telomere length (LTL) in newborns is 9.5 kb [10] whereas a length of ~5 kb was defined as the ‘telomeric brink’, which denotes a high risk of imminent death, but only 0.78% of people younger than 90 years display an LTL ≤ 5 kb [9]. So it is obvious, that the link between shortened telomeres and human longevity is more complex than mere reaching the critical telomere length. For instance, age-dependent telomere shortening might alter gene expression in sub-telomeric regions (telomere position effect, TPE) or double strand DNA breaks in telomeres might be inefficiently repaired and initiate cell senescence [11,12]. Furthermore, it has been suggested that even a single critically short telomere in a cell can induce cellular senescence, which potentially contributes to organismal senescence [13,14]. In humans, five short telomeres were reported to predict the onset of cell senescence [15].

Although the principles of protection and replication of telomeres are conserved and point to common evolutionary roots of eukaryotes, their implications for cell and organism survival, senescence, and aging are not shared among kingdoms. In particular, plants show specific features of their growth and development, which lead to confusion of terms like lifespan or aging as commonly used and understood in animals. First, a plant’s body plan is not fully established during embryogenesis and all tissues and organs are formed from proliferating meristem cells throughout the adult life. Second, plant growth is modular. Individual modules of the body (branches, flowers, leaves) are dispensable for survival, and their functions can be replaced by tissues newly differentiated from indefinitely proliferating meristems. This results in the enormous developmental plasticity of plants. Moreover, the vegetative meristems can give rise to a new organism, which will be a somatic clone, genetically indistinguishable from the parental organism. Since these general aspects distinguishing plant from animal development and aging have been well-reviewed [16], we will focus here on a more detailed view of peculiarities of plant telomere biology, including its latest developments.

## 2. Telomerase Core Components

**Figure 1:**
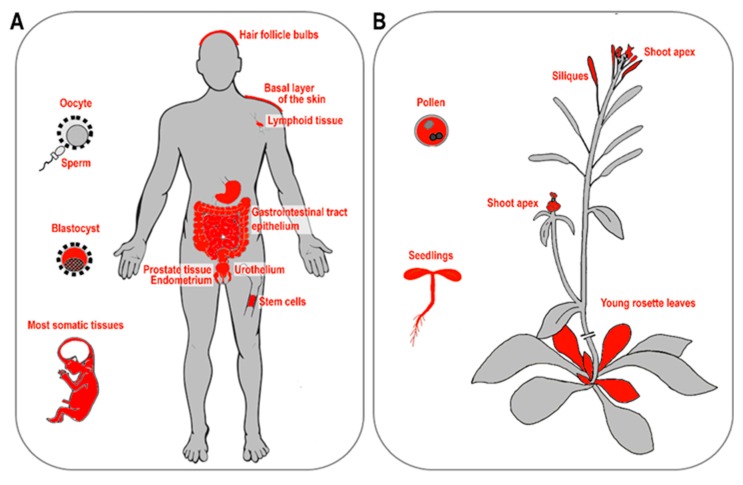
Telomerase activity in human and plant tissues. (**A**) During human embryonic development, high telomerase activity is detected in the blastocyst, but not in mature spermatozoa or oocytes. Highly active telomerase is detected in 16 to 20-week-old human fetuses in most somatic tissues with the exception of brain tissue [18,28]. In adults, low telomerase activity is detected in hair follicule bulbs [29], basal cells of crypt and villi or muconasal basal cells of the gastrointestinal tract, basal keratinocytes of the skin [30], lymphocytes, blood bone marrow, and stem cells [31,32,33], and urothelium [34]. High telomerase activity is detected in prostate tissues and endometrium [30,35]. (**B**) High telomerase activity is detected in plant pollen, seedling, young rosette leaves, and silliques [21,36,37,38,39]. Likewise, both apical meristems—shoot and root—show high telomerase activity [36,37,38]. Figures adopted from human and *Arabidopsis* eFP browsers [40].

The requirement to finish the incomplete replication of chromosome ends is common for all organisms with linear chromosomes. In eukaryotes, this requirement is commonly solved by a specific nucleoprotein enzyme complex called telomerase, which is considered as an ancestral telomere maintenance system that solves the end-replication problem of linear chromosomes. In humans, telomerase activity is detected in all early developmental stages from oocytes through to blastocyst stage embryos, and increases progressively with advancing embryo stage. Telomerase reaches its highest level in morula and blastocyst stage embryos and then decreases in the inner cell mass stage. In human fetuses—when the embryonic period and organogenesis are finished—telomerase is expressed in tissue-specific stem cells. However, just after birth, telomerase activity in somatic cells is downregulated with the exception of dividing cells (e.g., proliferating cells, T-lymphocytes) [17,18] (Figure 1A).

As seen in mammals, telomeres in plants are maintained by telomerase [19]. Active telomerase is detected in organs and tissues containing highly dividing meristem cells such as seedlings, root tips, young and middle-age leaves, flowers, and floral buds [20,21]. In terminally differentiated tissues (stems, mature leaves), telomerase activity is suppressed (Figure 1B). In some groups of organisms (in particular insects), telomerase has been lost and replaced by telomere-specific retrotransposons (in *Drosophila*) or tandem arrays of satellite repeats elongated by a gene conversion mechanism (reviewed in References [22,23]). Based on a long-term systematic search, no telomerase-independent exception has been found among vertebrates or land plants despite the variability of telomere DNA observed in land plants [24,25,26,27]. Besides the telomerase-based mechanism of telomere elongation, alternative lengthening of telomeres (ALT), which is based on homologous recombination (HR) and may become active upon the loss of telomerase was described in humans as well as in plants (see below). 

In yeasts, animals, and plants, telomerase consists of the telomerase reverse transcriptase (TERT) protein subunit providing the catalytic activity, and the telomerase RNA (TR) subunit whose short region provides a template for reverse transcription [41,42]. Besides these two core subunits, the telomerase complex comprises several other accessory proteins with diverse roles in telomerase assembly, trafficking, localization, recruitment to telomeres, or the processivity of telomere synthesis [43,44]. During movement of the maturing human telomerase complex through the nucleolus to Cajal bodies and to the telomeres, the TERT catalytical subunit is associated with e.g., HSP90, p23, or pontin. Assembly of human TR, as well as other box C/D or H/ACA small nucleolar RNAs (snoRNAs), is governed by conserved scaffold proteins: dyskerin, NHP2, NOP10, NAF1 in the nucleoplasm, where NAF1 is replaced by GAR1 before the hTR RNP complex reaches the nucleolus. Several orthologues of these conserved scaffold have been identified in plants, e.g., CBF5 (dyskerin), RuvBL1 (pontin), RuvBL2a (reptin), and NAF1. The nucleolar localization of these orthologues suggests potential conservation of the trafficking pathway during telomerase maturation ([45,46,47]; Schorova et al., submitted). Human and plant homologues of proteins associated either with the telomerase protein subunit TERT (Table 1) or the telomerase RNA subunit (Table 2) are listed below.

Considerable homology in TERT sequences and domain organization exists among organisms, and this homology has frequently been used to identify novel TERTs in genomic or transcriptomic data (reviewed in Reference [125]). Human TERT, as well as the plant TERTs, can be split into the N-terminal part, the central catalytic reverse transcriptase (RT) motifs, and the C-terminal extension (CTE) which is highly conserved among vertebrates as well as among plants. The N-terminal part comprises regions of both low and high similarity, e.g., the structural domains TEN (telomerase essential N-terminal domain) or TRBD (RNA-binding domain). Although most eukaryotes, including humans, harbor a single *TERT* gene, in the allotetraploid *Nicotiana tabacum* plant, three transcribed variants of the *TERT* gene were described, which were inherited from its diploid progenitor species [126].

Compared to the conserved structure of the TERT subunit, TRs show high sequence diversity among more distant organisms, as exemplified by the length differences of TRs in protozoa (159 nt in ciliate *Tetrahymena*, 2200 nt in *Plasmodium*), zebrafish (317 nt), mouse (397 nt), human (451 nt), and budding yeasts (1160 nt). Even within yeasts, the homology among TRs is rather low and their lengths range from 930 to more than 2000 nt [42,113,127,128,129,130,131,132,133]. Analogous variance of TR within the plant kingdom is still questionable, since only putative TRs have been predicted in *A. thaliana* so far [56].

However, several secondary structure motifs in TRs which are essential for telomerase activity are conserved in fungi and animals. Starting from the 5′-end of TR, these include a core-enclosing helix (CEH) formed by pairing the 5’-terminus of TR with the complementary internal TR region, a template boundary element (TBE)—a hairpin defining the end of the sequence recognized by TERT as a template, the template sequence itself, and a pseudoknot [133]. Except for the template sequence, none of these structural elements has been recognized in TER1 in *Arabidopsis thaliana*, which is the only reported candidate TR among plants so far [56]. With respect to the above-mentioned sequence diversity of plant telomere repeats, it will be interesting to learn whether and how these evolutionary changes are reflected by the corresponding TR subunits. For example, when assuming the phylogeny of Asparagales plants, telomeres switched first from *Arabidopsis*-like repeats (TTTAGGG)_n_ to human-like repeats (TTAGGG)_n_ in the divergence of the Iridaceae family, and this repeat survived all downstream speciation events until the divergence of the genus *Allium*, when the human-type repeat was replaced with the unusual (CTCGGTTATGGG)_n_ repeat [24,134,135]. The molecular basis underlying these evolutionary switches in telomere DNA sequences should be sought primarily in the corresponding TRs. We can consider the following possible scenarios. (i) TR remained essentially the same across Asparagales phylogeny and the observed switches in telomere synthesis occurred either as a result of mutations in the template region of TR or in its vicinity, which could have changed the boundaries of the region used as a template, (ii) a different RNA molecule took over the TR function. Experiments are in progress in our laboratory to provide a clear answer to this question.

## 3. Telomere Chromatin Composition

While the end-replication problem of telomeres is most commonly solved by telomerase, the other essential function of telomeres—their end-protection role (i.e., to distinguish natural chromosome ends from DNA breaks, and to eliminate unwanted repair events at telomeres)—is performed by other proteins associated with telomeres. In humans, these include proteins directly binding telomere DNA either in its double strand part (TRF1, TRF2) or at the single strand overhang (POT1). The other proteins bind telomeres via protein-protein interactions with these proteins (RAP1, TIN2, TPP1), which together form a complex termed shelterin [136,137]. Shelterin components and their interaction partners can inhibit the DNA damage response [138,139,140,141]. In addition to the end-protective function, shelterin components also play other roles as, e.g., the recruitment of telomerase to telomeres, facilitating replication fork movement through telomeres, or formation of telomere loops (t-loops) [142,143,144,145,146,147,148,149]. In particular, t-loops exist as a “closed-state” telomere conformation both in mammalians and plants [146,150]. While t-loop is considered as a structure inaccessible to telomerase, it may provide a template for telomerase-independent ALT (see below).

The composition of shelterin-like complexes shows differences in individual components among vertebrates, while the overall functions remain conserved. Human proteins associated with double and single strand telomeric DNA, together with their plant orthologues, are listed in Table 3 and Table 4, respectively.

In plants, knowledge of a shelterin-like complex is incomplete. The only proteins with confirmed in vivo telomere localization and function are members of the single-myb-histone family, telomere repeat binding (TRB) proteins, which have been characterised in *Arabidopsis thaliana* [66,82,151] and their orthologues were identified in other plants ([152]; Schorova et al., submitted). TRB proteins bind specifically telomeric double strand DNA through their myb-like domain of a telobox type [153,154], as well as the human core components of shelterin—TRF1 and TRF2 proteins. While the myb-like domain in TRF1 and TRF2 is localized at the C-terminus, that of TRB proteins occupies the N-terminus. Additionally, TRB proteins contain the centrally located histone-like domain (H1/5) involved in DNA sequence-unspecific DNA-protein interactions, multimerization, and interaction with POT1b (one of the plant POT1 paralogues) [65,151]. This plant-specific protein-domain organization has not been described in animals. TRB proteins bind telomeric DNA in vitro and in vivo, localize to the telomeres in vivo, interact directly with the telomerase TERT subunit, and the deregulation of telomeres was observed in mutant plants [66,68].

TRB proteins are not only components of the terminal complex associated with telomeres/telomerase, but they are also associated in vivo with promoters of translation machinery genes, which mostly contain a short telomeric sequence [67]. It seems that TRB proteins serve as epigenetic regulators that potentially affect the transcription status of thousands of genes by playing a role of recruiting subunits of multiple epigenetically active multi-protein complexes [68,69,70,71,155,156]. These findings are consistent with the observations from yeast or mammals where telomeric proteins (e.g., TRF1, TRF2, and RAP1) are able to localize outside telomeric regions and regulate the transcription of genes involved in metabolism, immunity, and differentiation [157,158,159,160,161,162,163,164].

Surprisingly, no functions in telomere maintenance were found in *Arabidopsis* orthologues of mammalian TRF proteins (TRFL proteins) where a myb-domain of the telobox type is located C-terminally as in human TRF1 and TRF2 [165]. However, a recent study revealed protein-protein interactions between TRFL2 and TRP1, members of the TRFL family, and TERT from *A. thaliana* [66,69]. Plant TRFL2 and TRP1 proteins interact with armadillo/β-catenin-like repeat-containing protein (ARM). ARM directly interacts with plant TERT [70] and might be involved in translation initiation or in regulation of recombination-related genes [69]. Moreover, ARM interacts with the chromatin remodeling protein CHR19 (Table 1). ARM, TRB1, POT1a, and CHR19 (but none of the TRFL proteins) were found among proteins that co-purified with *Arabidopsis* TERT using tandem affinity purification [84]. Association of TERT with proteins that are not essential for telomere maintenance may reflect possible non-telomeric functions of telomerase.

A dual function for telomerase, both telomeric and non-telomeric, is not unique to plants, as mammalian telomerase is involved not only in elongation of telomeres but also non-telomeric activities have been described, including involvement in regulating cellular processes such as apoptosis, proliferation, and cell cycle progression ([166]; reviewed in Reference [167]). Human telomerase and human ARM proteins play a role in the Wnt/APC/β-catenin signaling pathway [168]. A putative human homologue of ARM, ARMC6, interacts with the shelterin protein TRF2 and immuno-precipitates telomerase activity [69].

An additional telomere maintenance component is—somewhat paradoxically—Ku70/80 heterodimer, a DNA repair factor with a high affinity for DNA ends, that plays essential roles in the maintenance of genome integrity in both human and plants cells. In human cells, Ku70/80 heterodimer interacts with the RNA component of telomerase hTR [120] and with catalytic subunit hTERT [94]. In plants, Ku proteins, as well POT1b protein, are associated with TER2. This is a candidate plant TR that is not required for telomere maintenance in *A. thaliana* [56]. Ku70/80 is, however, important for protection of blunt-ended telomeres and for suppression of ALT (see below).

**Figure 2:**
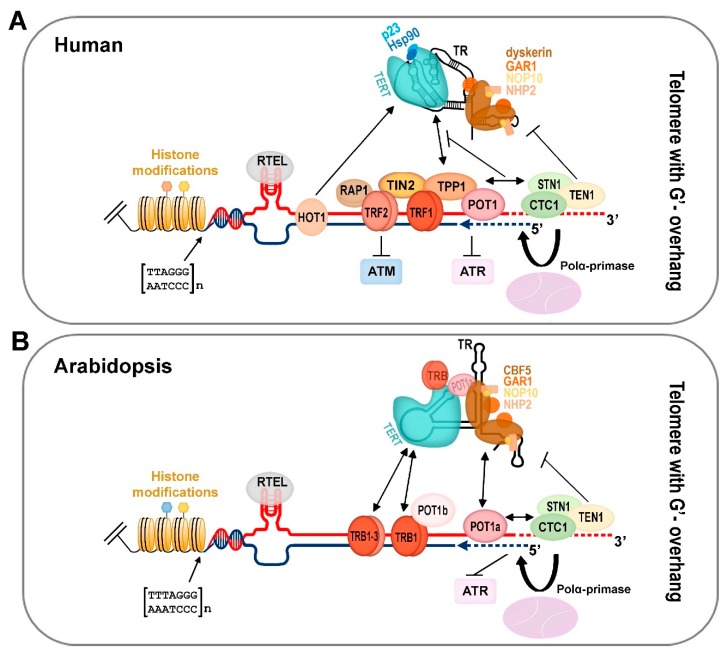
An integrative schematic view of the human and plant terminal telomeric complex. (**A**) Human active telomerase is associated with Hsp90 and p23 chaperones as well as with TR associated conserved scaffold proteins of box H/ACA small nucleolar RNAs (dyskerin, NHP2, NOP10, GAR1). Mammalian shelterin proteins (TRF1/2, RAP1, TIN2, TPP1, and POT1) modulate access to the telomerase complex and the ATR/ATM-dependent DNA damage response pathway. The CST complex (CTC1-STN1-TEN1) affects telomerase and DNA polymerase α recruitment to the chromosomal termini, and, thus, coordinates G-overhang extension by telomerase with fill-in synthesis of the complementary C-strand (blue dashed line). G-quadruplexes, D-loops, and t-loops during telomere replication are resolved by RTEL helicase. HOT1 directly binds double strand telomere repeats and associates with the active telomerase. Telomere nucleosomes show a shorter periodicity than that in the other parts of chromosomes. For human telomere histone modifications, see Figure 3. (**B**) *Arabidopsis* telomerase is associated with TRB proteins as well as with POT1a that interacts with the dyskerin orthologue CBF5. Plants possess all orthologue proteins of conserved scaffold box H/ACA of small nucleolar RNAs (CBF5, GAR1, NOP10, NHP2). Moreover, TRB proteins interact with the telomeric sequence due to the same myb-like binding domain as that in mammalian TRF1/2. TRB proteins interact with TERT and POT1b, and, when localized at chromosomal ends, they are eligible to function as components of the plant shelterin complex. An evolutionarily conserved CST complex is suggested to coordinate the unique requirements for efficient replication of telomeric DNA in plants as well as in other organisms. In addition, plant RTEL contributes to telomere homeostasis. For the sake of clarity, only the situation in telomere with 3′ overhang is depicted. For plant telomere histone modifications, see Figure 3.

An integrative updated schematic view based on these and previous studies is depicted in Figure 2. It is obvious that the number of plant telomere-associated and telomerase-associated orthologues (where they exist) is larger in comparison to their mammalian counterparts. The phenomenon of the multiplication of genes of the same family is not surprising, since in many plant families, polyploidy (i.e., whole genome duplication) resulting in retention of multiple gene paralogues may lead to their sub-functionalization, neo-functionalization, or partial or full redundancy [169,170]. In association with the previously mentioned evolutionary divergence of plant telomere DNA repeats toward human-like repeats or unusual telomeric repeats, it will be of interest to learn whether pre-existing components of plant shelterin-like complexes have adapted to the change in DNA sequence (this will be particularly interesting in proteins directly recognizing DNA sequences, such as the TRB or POT1 proteins), or whether some other proteins have replaced their function.

Besides the shelterin complex in mammals and its emerging equivalents in plants, there is yet another complex termed CST (CTC1-STN1-TEN1), which is involved in telomere maintenance. This tripartite complex binds the 3′-overhang of the G-rich strand of telomeric DNA and its function in telomere maintenance is conserved in both mammals and plants, and a similar complex exists also in yeast (with Cdc13 instead of CTC1 subunit) [171]. Recently, the roles of individual components of the human CST complex in telomere maintenance were elucidated: while CTC1-STN1 limits telomerase action to prevent G-overhang over-extension, TEN1 is essential for CST function in C-strand fill-in synthesis due to its stabilizing effect on binding the whole CST complex to telomeres and DNA polymerase α engagement in telomere synthesis [172,173]. CST functions, at least in humans, are not limited only to telomeres. CST is also required to avoid replication problem at G-rich sites throughout the genome, likely resolving replication fork stalling [174].

In addition to the telomere-specific proteins, the major part of telomeres is assembled into the nucleosomal chromatin structure which shows a shorter nucleosome periodicity (spacing) than that in the other parts of the chromosomes of the same organism [175,176,177,178,179]. Since shorter telomeres in cultured human cells show a lower nucleosome density than that in cells with longer telomeres, a close relationship was hypothesized between histone density, heterochromatin protein associations, telomere length, and TPE [180]. Interestingly, this feature of telomeric chromatin is conserved at least in vertebrates and plants, and may reflect the specific columnar structure of telomeric chromatin with stacked nucleosomes and weak determination of nucleosome positions by telomeric DNA sequence [181].

## 4. Telomere Epigenetics

As chromatin structures, telomeres are natural targets for epigenetic modifications. At the DNA level, methylation at carbon 5 of cytosine represents the dominant mark in eukaryotic cells. Methylated cytosines (^m^Cs) are generally enriched in heterochromatic regions of the genome and silenced promoters. Important differences in the sequence contexts, in which ^m^Cs are located, exist between animals and plants. In mammalian cells, they are predominantly located in CG doublet motifs, with the symmetry of the sequence crucial for the maintenance of the methylation pattern during DNA replication (reviewed in Reference [199]). A fraction of ^m^Cs in non-CG contexts was found in human embryonic cells. This fraction disappears after differentiation and is restored in induced pluripotent stem cells, which shows involvement of distinct methylation patterns in the regulation of gene expression [200]. Also in plants, cytosines in the CG motif are most frequently methylated, but ^m^Cs are also commonly placed in non-CG sequences, symmetrical CHG triplets (H=C or A or T), or non-symmetrical CHH motifs (reviewed in Reference [201]). In telomeres, cytosines in non-symmetrical sequence contexts are present in the telomeric C-rich strand, i.e., in CCCTAA repeats in animals and CCCTAAA repeats in plants. Using shotgun bisulfite genomic sequencing, ^m^Cs were detected in *A. thaliana* telomeric repeats with the inner cytosine most frequently methylated [202]. This pattern was confirmed by an independent approach, with high reliability at least in the proximal part of the telomere [203,204], and methylated telomeric cytosines were detected in cultured *Nicotiana tabacum* (tobacco) cells [205] and other plants [206]. Disruption of telomere homeostasis as a consequence of decreased genomic DNA methylation was observed in *A. thaliana* [203,207] but not in tobacco cells [205], which shows differences in the involvement of DNA methylation in regulation of telomere homeostasis between these model plants (for a more detailed review see Reference [208]).

Telomeres formed by mini-satellite repeats were traditionally considered as heterochromatic regions, and, thus, associated with heterochromatin-specific histone marks. Certain differences in histone modifications in heterochromatin have been described between animals and plants. In animals, constitutive heterochromatin is defined by the presence of H3K9me3 (trimethylation of lysine 9 of histone H3) (reviewed in Reference [209]) while in plants, this mark decorates silenced euchromatic genes, and constitutive heterochromatin is associated with H3K9me2 modification [210]. Facultative heterochromatin is enriched in H3K27me3 in cells of representatives of both kingdoms. In agreement with the hypothesis of the heterochromatic character of telomeres, the importance of heterochromatin-specific epigenetic marks for telomere maintenance and genome stability was demonstrated in numerous studies using human and mouse cells as models (reviewed in Reference [211]). On the other hand, data showing a low level of heterochromatin-specific modifications and an abundance of active marks on human telomeric histones have been presented [212,213,214], which shows certain dynamics of the human telomeric chromatin structure. Based on these and other reports, distinct differences exist in telomeric chromatin composition between the most important mammalian models, human and mouse cells, because H3K9me3 density and HP1 enrichment were significantly higher in mouse compared to humans [215,216]. Nevertheless, according to a study utilizing quantitative locus purification [217] the heterochromatic histone modification H4K20me3 is underrepresented at mouse telomeres even though it was previously detected by others at mouse [218,219] and also human [220] telomeres in analyses based on chromatin immuno-precipitation. Further research is necessary to draw final conclusions on the epigenetic nature of mammalian telomeres, especially considering other factors mentioned below.

Plant telomeric chromatin was shown to be associated with both heterochromatin-specific H3K9me2 and euchromatic H3K4me3 marks, with the latter less abundant [204,206,221]. Therefore, the plant telomeric chromatin exhibits a dual epigenetic character. Identification of the H3K27me3 modification, which is typical for facultative heterochromatin, in telomeric histones of *A. thaliana* [221,222] and *N. tabacum* [206] was rather surprising. However, it correlates with its presence at human telomeres [215], and with the recent observation that polycomb repressor complex 2-dependent loading of H3K27me3 at human telomeres is essential for the proper establishment of H3K9me3 and H4K20me3 modifications [220]. Nevertheless, H3K27me3 was not detected at mouse telomeres [217]. Thus, although significantly fewer results are available on the epigenetics of telomeric chromatin in plants compared to mammals, interesting similarities as well as differences have already been described and hopefully others will be elucidated based on future studies using different model organisms, including plants with non-canonical telomere sequences [24,25,27,134].

When discussing telomeric chromatin, it is necessary to mention that analysis of epigenetic modifications may be complicated by the presence of non-terminally located telomeric repeats forming interstitial telomeric sequences (ITSs). ITSs are relatively abundant in subtelomeric, pericentromeric, and centromeric regions of most eukaryotic organisms and represent fragile parts of chromosomes, which are prone to rearrangements and recombinations. The detailed compositions of telomeres and ITSs are different. In contrast to telomeres consisting of long tracts of perfect telomeric repeats, ITSs are often degenerated and/or disrupted by non-telomeric sequences. However, ITSs may still contribute to the telomere-specific signal in epigenetic studies, mainly those based on hybridization of membrane-bound DNA. Frequently-used genome-wide sequencing analyses (ChIP-seq and bisulfite sequencing) do not completely solve this problem because telomeres, like other tandem repeats, are difficult to analyze, and even direct analysis of respective read counts (i.e., those comprising perfect telomeric repeats versus those formed by degenerated repeats and non-telomeric sequences) may be ambiguous due to the non-linearity of PCR amplification of repetitive sequences [223]. Both mammalian and plant telomeres are transcribed to long non-coding RNA called TERRA [204,224] and this transcriptional potency could reflect the relatively lower level of compactness of telomeric chromatin compared with heterochromatin. The apparent discrepancy between the association of heterochromatic marks with telomeric histones and the transcriptional activity of telomeres is weakened by the facts that a mechanistic relationship between TERRA transcription and loading of heterochromatic modifications to human telomeres has been described [220], and that in *Arabidopsis* a certain—maybe dominant—fraction of TERRA is transcribed from ITSs [204], which are purely heterochromatic [225].

**Figure 3:**
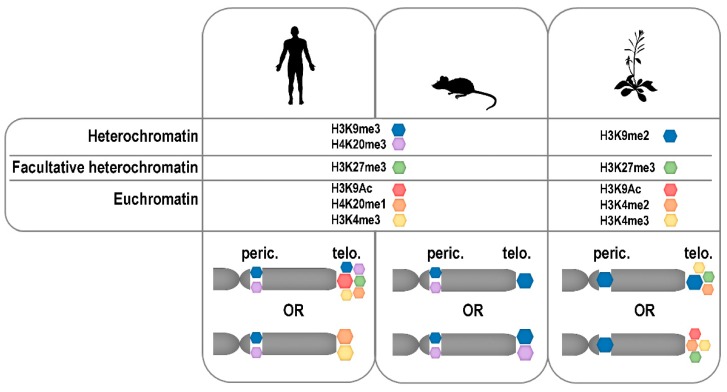
Modifications of mammalian and plant telomere (telo.) and pericentromere (peric.) histones. The relative enrichments of selected epigenetic modifications of telomeric and pericentromeric histones in human, mouse and *Arabidopsis* are schematically depicted according to data presented in References [204,212,213,215,217,218,219,220,221,222,225].

At this stage of knowledge, it is difficult or even impossible to formulate any general conclusion on the epigenetic nature of telomeric chromatin (Figure 3). Without any doubt, the specific structure of telomeres is crucial for the maintenance of genome integrity. Telomeres are rigid enough to prevent repair and recombination at chromosome ends and to restrict telomere accessibility for telomerase, but open enough to be transcribed and, at least in a specific time window of the cell cycle, accessible to telomerase. Moreover, in disagreements about telomeric “heterochromatin” or “euchromatin”, contribution of non-histone players, mainly shelterin proteins, to the telomeric chromatin compaction should be reflected (reviewed in Reference [226]). Why not admit, that telomeric chromatin is so specific that it does not fit into the existing criteria and that these should be widened? This suggestion is strengthened by the finding that other non-genic parts of the human genome, originally thought to be uniformly heterochromatic, are associated with different combinations of histone marks [213]. It is well possible that the epigenetic state of telomeres is more dynamic than previously thought and shows tissue-specific, cell-cycle specific, and developmental stage-specific changes. This would not only explain the diverse results of the above studies, but would be consistent with our current understanding of the epigenetics of other chromosome regions.

## 5. Telomere 3′-Overhangs, Blunt Ends, and Loops

Telomeres in vertebrates, in particular humans, possess 3′-overhangs at both chromosome ends. These overhangs are of different sizes on lagging versus leading strands [227]. In human telomeres a G-overhang is prevalent whose length varies from several tens to 280 nt [228,229,230]. Likewise, a 5′ C-rich overhang is present at the telomeres of human chromosomes, being far more prevalent in tumor cells using ALT (see below) [231]. This is not the case in *Arabidopsis thaliana*, *Silene latifolia*, and other angiosperm plants, which lack telomere overhangs or possess only short 1–3 nt overhangs at about half of their telomeres [232,233]. The telomere whose 3′- end is being synthesized in a given cell cycle by leading strand synthesis remains blunt-ended likely due to protection against end-processing by a specific exonuclease. This protection is dependent on the Ku70/80 heterodimer [233]. The role of the Ku complex in plant telomere protection was also suggested by our earlier studies, which indicated Ku as an interaction partner of AtTRP1, one of the TRF-like proteins in *A. thaliana* ([82]; see Reference [155] for a review). An analogous interaction between the shelterin components TRF2 and Ku70 was observed earlier in human cells [77]. Due to the asymmetry (non-equivalence) of plant telomeres, a different set of proteins may protect the telomere whose 3′-end serves as a template in “incomplete” lagging strand synthesis and can be elongated by telomerase. Protection of blunt-ended telomeres in *Arabidopsis* by the Ku70/80 complex seems paradoxical considering the presumed end-protective function of telomeres on one hand, and a key role of the Ku complex in non-homologous end-joining repair of double strand DNA breaks on the other hand. A possible solution of this enigma was suggested recently by a study which indicated different binding modes of the Ku complex to dsDNA breaks and to telomeres. Both functions were dissected using Ku mutants with impaired ability to translocate along DNA. While Ku sliding is not required for its association with plant telomeres, it is essential for its involvement in the non-homologous end joining pathway of DNA repair [101]. The presence of blunt-ended telomeres is, however, not common to all plants. For example, in the moss *Physcomitrella patens*, both telomeres of a chromosome possess overhangs and, correspondingly, lack of the Ku complex components shows no effect on telomere maintenance or end protection [234]. The Ku70/80 complex was also reported to be a negative regulator of telomerase function in *Arabidopsis* [99]. In addition to telomere elongation by telomerase, an extension of telomere G-strand overhangs was observed in Ku mutants, which suggests a role of Ku70/80 in C-rich telomeric strand maintenance [235].

Besides telomerase, eukaryotic cells can also utilize a back-up mechanism of telomere maintenance—ALT—which is based on homologous recombination (HR) [236]. This telomerase-independent mechanism is activated in a number of human tumors, in human cells immortalized in culture, and also in normal somatic tissues [237]. In plants, the ALT mechanism is activated in mutants with telomerase dysfunction and possibly also during the earliest stages of normal plant development [238]. ALT relies on the formation of terminal telomeric loops (t-loops) [146], which parallels the first steps of HR. The eventual resolution of these t-loops and aberrant HR at telomeres generates not only telomeres of highly heterogeneous lengths but also extrachromosomal t-circles, which are the known hallmarks of ALT. In mutant plants that are deficient for components of the Ku70/80 complex, induction of t-circle formation was observed at telomeres but not at other regions rich in DNA repeats. Despite ongoing terminal deletions arising from excision of t-circles in mutant plants, the telomeres remain functional, which indicates an efficient telomere healing by telomerase [239].

Another interesting protein connecting telomeric loops and circles with DNA recombination and telomere replication is RTEL1. This was originally described in *Caenorhabditis elegans* as a functional homologue of the yeast Srs2 protein, which removes Rad51 from single strand DNA. Therefore, it prevents the homology search step of HR and helps to protect the cell from inappropriate HR (for review, see Reference [240]). Furthermore, in *C. elegans*, the RTEL1 helicase suppresses inappropriate recombination events by promoting disassembly of D-loop recombination intermediates, and the loss of its function results in increased genome instability [241]. In addition to its regulatory role in HR, RTEL1 acts in telomere maintenance in mammalian telomerase-positive cells [242]. This function was explained by the function of RTEL1 in opening t-loops, which blocked inappropriate excision of large telomere regions—the process known as telomere rapid deletion. To promote this t-loop unwinding, RTEL1 is recruited to telomeres in the S-phase by the telomeric protein TRF2 [186].

In addition to its role in t-loop stability, mouse RTEL1 can dissolve G4-DNA structures, which otherwise block replication fork progression and the extension of telomeres by telomerase [243]. Importantly, the role of RTEL1 in telomere dynamics was clearly confirmed by the finding that its mutation is causative for Hoyeraal-Hreidarsson syndrome, which is a severe form of dyskeratosis congenita, predisposing to bone-marrow failure and cancer. This disease is characterised by short telomeres and genome instability [244,245,246]. A recent report revealed that reversed replication forks occurring in telomeres of RTEL1-deficient cells is due to compromised telomere replication aberrantly recruiting telomerase, which prevents the restart of reversed replication forks at telomeres and leads to critically short telomeres [247]. In this context, telomerase paradoxically contributes to telomere shortening by stabilizing stalled replication forks at chromosome ends.

In addition, the *A. thaliana* RTEL1 homolog suppresses HR and is involved in processing DNA replication intermediates and interstrand and intrastrand DNA cross-links. Deficiency of the *Arabidopsis* RTEL1 triggers a SOG1-dependent replication checkpoint in response to DNA crosslinks [248]. Similarly to the situation in mammals, the *Arabidopsis* RTEL1 contributes to telomere homeostasis. The concurrent loss of RTEL1 and TERT accelerates telomere shortening, which results in a developmental arrest after four generations [249] compared to 10 generations in single-mutant *tert* plants [250]. This observation indicates a role of RTEL1 in ALT, which otherwise partially compensates for the loss of TERT [238]. In agreement with these results, it was recently demonstrated that RAD51-dependent homologous recombination participates in ALT in *A. thaliana* [251]. This is not surprising when considering the essential role of RAD51 in HR, and HR as a major molecular mechanism of ALT. However, the authors further showed that this role of RAD51 is dependent on RTEL1 helicase, which possibly functions in dissolution of the D-loop after telomere replication. In P. patens, RTEL1 has been found among genes, which are up-regulated after γ-irradiation. RTEL1 knockout resulted in a severe growth deficiency, which was independent of the presence of bleomycin [252], and the authors hypothesized that this growth phenotype might be the result of telomere deficiency. Thus, the functions of RTEL1 seem widely conserved. In conclusion, the requirement for RTEL1 in multiple pathways to preserve plant genome stability can be explained by its putative role in the destabilization of DNA loop structures such as D-loops and t-loops, which aligns with previous studies in mammalian systems.

## 6. Cellular Aging and the Immortal DNA Strand Hypothesis

Cellular aging is characterized by progressive loss of physiological integrity that leads to impaired function and genomic instability and ultimately to a functional decline at the tissue and organ level. Telomere attrition during cell aging is classified as one of the several major hallmarks of aging—together with, e.g., genomic instability, epigenetic alterations, loss of proteostasis, mitochondrial dysfunction, cellular senescence, or altered intercellular communication [7]. In Metazoa, there is no universal pattern of telomere erosion [253], and, in some animals, the progressive telomere shortening with age has not been observed [254]. Nevertheless, telomere length is typically inversely correlated with lifespan, while telomerase expression co-evolved with body size [255]. A connection between cellular aging and replicative telomere shortening is widely accepted and experimentally validated in both humans and plants. Importantly, under normal conditions (in wild type plants) this type of cellular aging is prevented by telomerase activity in dividing cells [20,21,38]. The associations between telomere length and age-related disease and mortality in humans have been proven in several studies (reviewed in References [8,256,257]). However, telomere length of humans is not a determinant of aging but rather a marker able to explain life expectancy and disease risk.

In animals, the distribution of cellular age varies among tissues and cell compartments, including progenitor cell compartments, depending on the influx of stem cells and the dynamics of self-renewal and differentiation of progenitor cells. In particular, the mode of cell division of progenitor cells may be: (i) symmetric self-renewal, in which progenitor cell division results in two daughter progenitor cells (one generation older) remaining in the compartment, (ii) symmetric differentiation, resulting in two differentiated cells which leave the progenitor cell compartment, or (iii) asymmetric division resulting in one progenitor and one differentiated cell. Importantly, cellular age distributions between healthy and cancerous tissues may inform dynamic changes within the hierarchical tissue structure, i.e., an acquired increased self-renewal capacity in certain tumors [258]. In this connection, it is of interest to mention the hypothesis of the immortal DNA strand [259]. This hypothesis proposes that adult stem cells segregate their template and newly synthesized DNA strands non-randomly, preferentially retaining parental DNA strands in each division. This way, adult stem cells pass mutations resulting from replication errors onto non-stem cell daughter cells that differentiate and terminate division. Adult stem cells could thus reduce the accumulation of mutations and the associated deterioration of gene functions with each cell cycle. Moreover, this strategy would also slow down replicative telomere shortening. Thus, two major factors of cellular and organismal aging could be substantially limited if immortal DNA strand segregation operates in progenitor cells. Several studies have supported this hypothesis up to now. For example, using sequential pulses of halogenated thymidine analogues, high frequencies of segregation of older and younger template strands during proliferative expansion of mouse muscle stem cells was observed [260]. Template strand co-segregation was strongly associated with asymmetric cell divisions yielding daughters with divergent fates. Daughter cells inheriting the older templates retained a more immature phenotype, whereas daughters inheriting the newer templates acquired a more differentiated phenotype. It will be of interest to learn if the validity of this hypothesis is more general, and specifically to elucidate the molecular mechanism of non-random DNA segregation in asymmetric cell division. This principle may also be functional in meristem cell division and differentiation. While replicative telomere shortening is efficiently counteracted by telomerase in wild type plants (see above), reduction of accumulation of mutations would be extremely beneficial when considering e.g., trees sustaining their growth for centuries. Low telomere loss per plant generation has been found in telomerase-deficient *Arabidopsis* mutants [250], which indicates a possible involvement of non-random DNA strand segregation in addition to ALT [238]. Unfortunately, the application of sequential pulse labeling in planta is technically too demanding, and any direct evidence for the immortal DNA strand hypothesis is, thus, missing in plants.

## 7. Concluding Remarks

Currently available data show remarkably conserved principles in telomere biology across eukaryotes, which is consistent with an association of telomere and telomerase emergence with the earliest steps of their evolution. At the same time, however, a number of specific features and exceptions cannot be ignored since they point to limitations of our wider understanding of these principles. Among a number of open questions to be answered, elucidation of the structure of telomeric chromatin (telochromatin), including its epigenetic and higher-order dynamics, with high spatial and temporal resolution is needed in various model systems. Furthermore, the biological relevance of non-canonical structures formed by telomeric DNA should be addressed mainly under in vivo conditions. Such studies are timely due to recent fast progress in adequate technical tools, including e.g., super-resolution and cryo-electron microscopy.

Studies of repair processes at telomeres and of telomerase regulation belong to the hot topics in this field, since this knowledge can clearly be applied to promote protection of genome stability. In this respect, plants are indispensable due to the natural telomerase-competent character of their cells which allows us to examine mechanisms of repression and activation of telomerase in association with proliferation, differentiation, and dedifferentiation of plant cells. This knowledge is essential for understanding carcinogenesis and is potentially applicable to tumor therapy and cell rejuvenation.

## Figures and Tables

**Table 1:** Comparative overview of proteins associated with the telomerase catalytic subunit TERT.

Telomerase Calytic Subunit (TERT) Associated Proteins.					
Human TERT Associated Proteins	Protein Function and Direct Interactions	References	*Arabidopsis* TERT Associated Proteins	Protein Function and Direct Interactions	References
**TERT**	**Catalytic subunit of telomerase**	**[48]**	**TERT**	**Catalytic subunit of telomerase**	**[49]**
**POT1**	Shelterin. Int.: telomeric ssDNA, TPP1 and CTC1.	[50,51,52,53,54]	**POT1a**	Shelterin-like. Int.: TERT, telomeric ssDNA, TER1, TRFL9, CBF5, RuvBL1, CTC1 and STN1.	[47,55,56,57,58]
**TRF1**	Shelterin. Int.: telomeric dsDNA, TIN2, TANK1, PINX1, and ATM.	[59,60,61,62,63]	**TRB1-3**	Shelterin-like. Int.: TERT, telomeric dsDNA, POT1b, RuvBL1 and RuvBL2a.	[64,65,66,67,68,69,70,71]
**TRF2**	Shelterin. Int.: telomeric dsDNA; TIN2, NBS1, RAD50, Apollo, Ku70, PARP1, XPF-ERCC1, BLM, FEN1, POLB, ORC, RTEL1, ATM and HP1.	[61,72,73,74,75,76,77,78,79,80]	**TRP1**	Possible non-telomeric functions of telomerase. Int.: TERT, telomere dsDNA in vitro, ARM, Ku70 and TRFL9.	[66,69,81,82,83]
			**TRFL2**	Possible non-telomeric functions of telomerase. Int.: TERT, telomere dsDNA in vitro and ARM.	[69,83]
			**TRFL11**	Associates with TERT.	[84]
**KPNA1**	Promotes nuclear import of the TERT.	[85]	**ImpA4**	Associates with TERT.	[84]
**NCL**	Involves nucleolar localization of TERT.	[86]	**NUC-L1**	Role in telomere maintenance and telomere clustering.	[87,88]
**pontin**	Telomerase assembly. Int.: TERT and dyskerin.	[89]	**RuvBL1**	Associates with TERT via TRBs, regulates telomerase activity.	[84,90]
**reptin**	Telomerase assembly. Int.: dyskerin.	[89]	**RuvBL2a**	Associates with TERT via TRBs, regulates telomerase activity.	[84]
**ARMC6**	Int.: TRF2, telomerase.	[69,91]	**ARM**	May reflect posible non-telomeric functions of telomerase. Int.: TERT, TRP1, TRFL2, TRFL9 and CHR19.	[69,92]
**TPP1**	Shelterin, mediates telomerase recruitment. Int.: TERT, POT1, TIN2, CTC1 and STN1.	[51,52,53,54,75]	**n.a.**		
**PINX1**	Potent telomerase inhibitor. Int.: TERT and TRF1.	[62]	**n.a.**		
**HOT1**	Int.: telomeric dsDNA, active telomerase.	[93]	**n.a.**		
**Ku70/80**	Int.: TERT, TR, TRF2 and RAP1.	[94,95]	**Ku70/80**	Role in telomere length regulation, may protect blunt-ended telomeres Int.: TRP1, TER2 and TER2s.	[82,96,97,98,99,100,101]
**Hsp90**	TERT assembly. Int.: TERT.	[102]	**Hsp90**	NP_194150.1	[103]
**p23**	TERT assembly. Int.: TERT.	[102]	**p23**	CAC16575, NP_683525	[104]
**Purα**	p.h. Unwinds dsDNA telomeric oligonucleotides.	[105]	**PURα1**	Associates with TERT.	[84]
**SMARCAD1**	p.h. SWI/SNF-like protein that presumably associates with telomeres.	[106,107]	**CHR19**	May reflect possible non-telomeric functions of telomerase. Int.: TERT, ARM, TRB1 and TRFL9.	[69]
**PABPN1**	Promotes poly(A)-dependent TR 3′ end maturation.	[108]	**RRM**	Associates with TERT.	[92,109]
**MT2A**	p.h. Int.: HOT1.	[110,111]	**MT2A**	Associates with TERT.	[84,109]
**PA2G4**	NP_006182.2	[112]	**G2p**	Associates with TERT.	[84,109]

The proteins depicted in grey are involved in telomere maintenance, however, their association with telomerase has not been described. The proteins in green are structural homologous to their human/plant counterparts, however, any involvement in telomere maintenance or association with telomerase has not been described so far. Direct interaction partners (Int.) of TERT-associated proteins are enumerated. Cases with not yet identified sequence homologues are denoted with n.a. ATP-dependent DNA helicase 2 subunit 1 and 2 (Ku70/80); Origin recognition complex (ORC); RuvB-like 2 (reptin); TIN2- and POT1-organizing protein (TPP1); TRF1-interacting nuclear protein 2 (TIN2); TRF1-interacting protein 1 (PINX1); 5′ exonuclease Apollo (Apollo); Armadillo repeat-containing protein 6 (ARMC6); Armadillo/β-catenin-like repeat-containing protein (ARM); Ataxia telangiectasia mutated kinase (ATM); Bloom syndrome protein (BLM); Centromere-binding factor (CBF5); Conserved telomere maintenance component 1 (CTC1); DNA polymerase beta (POLB); DNA repair protein RAD50 (RAD50); Double strand DNA (dsDNA); Excision repair cross-complementation 1 (ERCC1); Flap endonuclease 1 (FEN1); H/ACA ribonucleoprotein complex subunit DKC1 (dyskerin); Heterochromatin protein 1 (HP1); Homeobox telomere-binding protein 1 (HOT1); Hsp90 co-chaperone (p23); Chromatin remodeling 19 (CHR19); Importin-α5 (KPNA1); Importin subunit alpha-4 (ImpA4); Metallothionein-like 2A (MT2A); Nijmegen breakage syndrome protein 1 (NBS1); Nucleolin (NCL); Nucleolin like 1 (NUC-L1); Heat shock protein HSP 90 (Hsp90); Poly(ADP-ribose) polymerase 1 (PARP1); Polyadenylate-binding protein (PABPN1); Proliferation-associated 2G4 (PA2G4); Proliferation-associated protein (G2p); Protection of telomeres 1 (POT1); Protection of telomeres 1a, b (POT1a, b); Pur-alpha 1 (Purα1); Regulator of telomere elongation helicase 1 (RTEL1); RNA recognition motif (RRM); RuvB-like 1 (pontin); RuvB-like 1, 2a (RuvBL1, 2a); Single strand DNA (ssDNA); Suppressor of cdc thirteen homolog (STN1); SWI/SNF-related matrix-associated actin-dependent regulator of chromatin subfamily A containing DEAD/H box 1 (SMARCAD1); Tankyrase 1 (TANK1); Telomerase reverse transcriptase (TERT); Telomerase RNA (TR); Telomerase RNA subunit 1 (TER1); Telomere repeat-binding factor 1, 2, 3 (TRB1, 2, 3); Telomere repeat-binding protein 1 (TRP1); Telomeric repeat binding factor 1-like 2, 9, 11 (TRFL 2, 9, 11); Telomeric repeat-binding factor 1, 2 (TRF1, 2); Xeroderma pigmentosum group F (XPF1); putative homolog according to NCBI blastp (p.h.).

**Table 2:** Comparative overview of proteins associated with the RNA component of telomerase.

Telomerase RNA Associated Proteins					
Human TR Associated Proteins	Protein Function and Direct Interactions	References	*Arabidopsis* TR Associated Proteins	Protein Function and Direct Interactions	References
**TR**	**RNA subunit of telomerase**	**[113]**	**TER1, TER2, TER2s**	**Putative RNA subunit of telomerase**	**[56,100]**
**TERT**	Catalytic subunit of telomerase	[48,114]	**TERT**	Catalytic subunit of telomerase	[100]
**Dyskerin**	H/ACA snoRNPs, associated with nucleolus. Int.: TR, GAR1, NHP2, NOP10 and TCAB1.	[44,115]	**CBF5**	H/ACA snoRNPs, Ath orthologue of Dyskerin, associated with nucleolus, subnuclear bodies and Cajal bodies, associated with telomerase RNP complex. Direct interaction with either of putative TERs not demonstrated. Int.: NAF1.	[45,57]
**NOP10**	H/ACA snoRNPs, associates with nucleolus. Int.: TR and dyskerin.	[44,116]	**NOP10**	H/ACA snoRNPs, Ath orthologue of NOP10, associates with nucleolus.	[45,46]
**NHP2**	H/ACA snoRNPs, associates with nucleolus. Int.: TR, dyskerin and TCAB1.	[117,118]	**NHP2**	H/ACA snoRNPs, Ath orthologue of NHP2, associates with nucleolus.	[45,46]
**GAR1**	H/ACA snoRNPs, associated with nucleolus. Int.: dyskerin and TCAB1.	[44,118]	**GAR1, 2**	H/ACA snoRNPs, Ath orthologues of GAR1, associate with nucleolus.	[45,46]
**NAF1**	H/ACA snoRNPs, nucleolar shuttle - NAF1 is substituted by GAR1 during maturation of telomerase. Int.: dyskerin.	[119]	**NAF1**	H/ACA snoRNPs, Ath orthologue of NAF1, associates with nucleolus and Cajal bodies. Int.: CBF5.	[45]
**Ku70/80**	Int.: TR, TERT, TRF2 and RAP1.	[95,120]	**Ku70/80**	Role in telomere length regulation, may protect blunt-ended telomeres Int.: TRP1, TER2 and TER2s.	[100]
**pontin**	Telomerase assembly. Int.: TERT and dyskerin.	[89]	**RuvBL1**	Associates with TERT via TRBs, regulates telomerase activity.	Schorova et al., submitted
**reptin**	Telomerase assembly. Int.: dyskerin.	[89]	**RuvBL2a**	Associates with TERT via TRBs, regulates telomerase activity.	Schorova et al., submitted
**RHAU**	RNA helicase, unwinds a G4-quadruplex in human telomerase RNA. Int.: TR.	[121]	**RHAU**	NP_850255.1, NP_175298.2, NP_680142.2, NP_178223.2	n.a.
**PARN**	Poly(A)-specific ribonuclease, 3′-end maturation of the TR. Int.: TR	[122]	**PARN**	Poly(A) degradation activity, essential gene first required during early development.	[123]
**TCAB1**	H/ACA snoRNPs, driving telomerase to Cajal bodies. Int.: TR, dyskerin, NHP2 and GAR1.	[124]	**TCAB1**	NP_193883.2	n.a.

The proteins in green are structural homologues to their human counterparts, however, any involvement in telomere maintenance or association with RNA component of telomerase has not been described so far. Direct interaction partners (Int.) of TR-associated proteins are enumerated. Cases when reference is not available are denoted n.a. H/ACA ribonucleoprotein complex subunit DKC1 (dyskerin); RuvB-like 2 (reptin); *Arabidopsis* (Ath); ATP-dependent DNA helicase 2 subunit 1 and 2 (Ku70/80); box H/ACA small nucleolar RNA-protein complexes (H/ACA snoRNPs); Centromere-binding factor (CBF5); Glycine arginine rich 1, 2 (GAR1, 2); Non-histone protein 2 (NHP2); Nuclear assembly factor 1 (NAF1); Nucleolar protein 10 (NOP10); Repressor-activator protein 1 (RAP1); RNA helicase (PARN); RNA helicase (RHAU); RuvB-like 1 (pontin); RuvB-like 1, 2a (RuvBL1, 2a); Telomerase Cajal body protein 1 (TCAB1); Telomerase reverse transcriptase (TERT); Telomere repeat-binding factors (TRBs); Telomere repeat-binding protein 1 (TRP1); Telomerase RNA subunit 1, 2, 2s (TER1, 2, 2s); Telomeric repeat-binding factor 2 (TRF2); Telomerase RNA (TR).

**Table 3:** Comparative overview of proteins associated with telomeric double strand DNA (dsDNA).

Telomeric dsDNA Associated Proteins					
Human Telomeric dsDNA Associated Proteins	Protein Function and Direct Interactions	References	*Arabidopsis* Telomeric dsDNA Associated Proteins	Protein Function and Direct Interactions	References
**TRF1**	Shelterin. Int.: telomeric dsDNA, TIN2, TANK1 and PINX1. Non-telomeric: binding to ITS and chromatin and satellite DNA and modulation of their chromatin structure. Control of a common fragile site containing ITS.	[59,60,61,62] [162,182]	**TRB1, 2, 3**	Shelterin-like. Int.: telomeric dsDNA, TERT, POT1b, RuvBL1 and RuvBL2a. Non-telomeric functions - a recruitment subunit of protein complexes involved in epigenetic regulations. Binding to ITSs.	[64,65,66]; Schorova et al., submitted [67,68,69,70,71]
**TRF2**	Shelterin. Int.: telomeric dsDNA; TIN2, RAP1, NBS1, RAD50, Apollo, Ku70, PARP1, XPF-ERCC1, BLM, FEN1, POLB, ORC, RTEL1 and ATM.	[61,72,73,74,75,76,77,78,79,80,183,184,185,186,187]	**TRP1**	Possible non-telomeric functions of telomerase. Int.: telomere dsDNA in vitro, TERT, ARM, Ku70, TRFL1 and TRFL9.	[66,69,81,82,83]
	Non-telomeric function: transcriptional regulator. Binding to ITSs and satellite III.	[155,163]	**TRFL2**	Possible non-telomeric functions of telomerase. Int.: telomere dsDNA in vitro, TERT and ARM.	[69,83]
			**TRFL9**	Possible non-telomeric functions of telomerase. Int.: telomere dsDNA in vitro, TRP1 and POT1a.	[69,83]
			**TBP1, TRFL1, TRFL4**	Int.: telomere dsDNA in vitro.	[83,188]
**HOT1**	Int.: telomeric dsDNA, active telomerase.	[93]	**n.a.**		
**Ku70/80**	The way of association with telomeric dsDNA is not fully elucidated. Int.: TRF2, RAP1, TR and TERT.	[95]	**Ku70/80**	Role in telomere length regulation, may protect blunt-ended telomeres Int.: TRP1, TER2 and TER2s.	[82,96,97,98,99,101]

The proteins depicted in grey are involved in telomere maintenance, however, their association with telomeric dsDNA has not been fully proven yet. Direct interaction partners (Int.) interacting with telomeric dsDNA-associated proteins and concerning their telomeric functions are enumerated. No sequence homologue has been identified yet (n.a.). Double-strand DNA (dsDNA); 5′ exonuclease Apollo (Apollo); Armadillo/β-catenin-like repeat-containing protein (ARM); Ataxia telangiectasia mutated kinase (ATM); ATP-dependent DNA helicase 2 subunit 1 and 2 (Ku70/80); Bloom syndrome protein (BLM); DNA polymerase beta (POLB); DNA repair protein RAD50 (RAD50); Excision repair cross-complementation 1 (ERCC1); Flap endonuclease 1 (FEN1); Homeobox telomere-binding protein 1 (HOT1); Interstitial telomeric sequences (ITSs); Nijmegen breakage syndrome protein 1 (NBS1); Origin recognition complex (ORC); Poly(ADP-Ribose); polymerase 1 (PARP1); Protection of telomeres 1b (POT1b); Regulator of telomere elongation helicase 1 (RTEL1); Repressor-activator protein 1 (RAP1); Telomerase RNA (TR); RuvB-like 1, 2a (RuvBL1, 2a); Tankyrase 1 (TANK1); Telomerase reverse transcriptase (TERT); Telomerase RNA subunit 2, 2s (TER2, TER2s); Telomere binding protein 1 (TBP1); Telomere repeat-binding factor 1, 2, 3 (TRB1, 2, 3); Telomere repeat-binding protein 1 (TRP1); Telomeric repeat binding Factor 1-like 1, 2, 4, 9 (TRFL1, 2, 4, 9); Telomeric repeat-binding factor 1 (TRF1); Telomeric repeat-binding factor 2 (TRF2); TRF1-interacting nuclear protein 2 (TIN2); TRF1-interacting protein 1 (PINX1); Xeroderma pigmentosum group F (XPF1).

**Table 4:** Comparative overview of proteins associated with telomeric single strand (ssDNA).

Telomeric ssDNA Associated Proteins					
Human Telomeric ssDNA Associated Proteins	Protein Function and Direct Interactions	References	*Arabidopsis* Telomeric ssDNA Associated Proteins	Protein Function and Direct Interactions	References
**POT1**	Shelterin. Int.: telomeric ssDNA, TPP1 and CTC1.	[50,51,52,53,54]	**POT1a**	Shelterin-like. Int.: TERT, telomeric ssDNA, TER1, TRFL9, CBF5, RuvBL1, CTC1 and STN1.	[47,55,56,57,58,69,105,189]
			**POT1b**	Shelterin-like. Int.: TRB1, TER2, TER2s.	[56,82,100]
			**POT1c**	POT1 paralogue of unknown function.	[47]
**TERT**	Catalytic subunit of telomerase.	[190]	**TERT**	Catalytic subunit of telomerase.	
**STN1**	CST complex subunit, prevents G-overhang overextension. Int.: CTC1, TEN1, TPP1 and POLA.	[54,172,191,192]	**STN1**	CST complex subunit, controls access of telomerase and DDR, together with POLA may be involved in C-strand synthesis. Int.: CTC1, TEN1 and POT1a. Non-telomeric function. Facilitates re-replication at non-telomeric loci.	[189,193,194,195]
**TEN1**	CST complex subunit, involves C-strand fill-in synthesis. Int.: STN1.	[172,192]	**TEN1**	CST complex subunit, controls access of telomerase and DDR, coordinating synthesis of the C-strand. Int.: STN1.	[194]
**CTC1**	CST complex subunit, prevents G-overhang overextension. Int.: telomeric ssDNA, STN1, TPP1 and POT1.	[54,192]	**CTC1**	CST complex subunit, controls access of the telomerase and DDR, coordinating synthesis of the C-strand. Int.: STN1, POT1a and POLA.	[171,189,196]
**Purα**	p.h. Unwinds dsDNA telomeric oligonucleotides.	[105]	**PURα1**	Associates with TERT.	[84]
**n.a.**			**Why1**	Regulates telomere-length homeostasis. Int.: telomeric ssDNA.	[197]
**n.a.**			**STEP1**	Truncated derivative of chloroplast RNA-binding protein, role in plant telomere biogenesis. Int.: telomeric ssDNA.	[198]

The proteins depicted in grey are involved in telomere maintenance, however, their association with telomeric ssDNA has not been fully proven yet. The proteins in green are structural homologues of their human/plant counterparts, however, any involvement in telomere maintenance or association with telomeric sequences has not been described so far. Direct interaction partners (Int.) interacting with telomeric ssDNA associated proteins are enumerated. Cases with not yet identified sequence homologues are denoted with n.a. Single strand DNA (ssDNA); Double-strand DNA (dsDNA); Cajal bodies factor 5 (CBF5); Conserved telomere maintenance component 1 (CTC1); CST complex (CTC1, STN1 and TEN1 subunits); DNA damage response (DDR); DNA polymerase alpha (POLA); Protection of telomeres 1 (POT1); Protection of telomeres 1a, b, c (POT1a, b, c); Pur-alpha 1 (Purα1); RuvB-like 1 (RuvBL1); Single-stranded telomere-binding protein 1 (STEP1); Suppressor of cdc thirteen homolog (STN1); Telomerase reverse transcriptase (TERT); Telomerase RNA subunit 1, 2, 2s (TER1, 2, 2s); Telomeric pathways in association with STN1 (TEN1); Telomeric repeat binding factor 1 -like 9 (TRFL9); TIN2- and POT1-organizing protein (TPP1); Whirly 1 (Why1); putative homolog according to NCBI blastp (p.h.).
